# Quantification of the increase in the frequency of early calving associated with late exposure to bluetongue virus serotype 8 in dairy cows: implications for syndromic surveillance

**DOI:** 10.1186/s13567-015-0296-7

**Published:** 2016-01-13

**Authors:** Simon Nusinovici, Aurélien Madouasse, Christine Fourichon

**Affiliations:** INRA, UMR1300 Biology, Epidemiology and Risk Analysis in Animal Health, CS 40706, F-44307 Nantes, France; LUNAM Université, Oniris, UMR1300 Biology, Epidemiology and Risk Analysis in Animal Health, CS 40706, F-44307 Nantes, France

## Abstract

A recent study evaluating whether reproductive data could be used for syndromic surveillance found an increased frequency of early calving (calving occurring a few days earlier than expected) in areas exposed to the Bluetongue virus serotype 8 (BTV-8) in northern Europe. A high proportion of herds infected during the 2006–2009 European outbreak were not reported through the surveillance system. The objectives of this study were (1) to quantify the increase in the frequency of early calving associated with the exposure to BTV-8 in late gestation and (2) to determine whether this association could be found in populations exposed to BTV-8 but without reported clinical signs. Increases in frequency of early calving were quantified for cows in herds located in the 2007 outbreak area in France, reported or not as cases. Increases were detected for cows in both categories of herds with a larger effect in herds reported after clinical signs. Moreover, the largest effect was found for exposures occurring during the latest stage of pregnancy, suggesting that BTV infection could trigger calving in cows in late gestation, a few days earlier than expected. This is the first study quantifying the association between a viral infection and a shortened pregnancy length (still within a normal range). The high magnitude of the increase in frequency of early calving, their occurrence in herds from infected areas but not reported, and the short time interval between exposure and the occurrence of the event confirm the interest of using early calving as an indicator for syndromic surveillance.

**Electronic supplementary material:**

The online version of this article (doi:10.1186/s13567-015-0296-7) contains supplementary material, which is available to authorized users.

## Introduction

The number of infectious diseases to emerge in humans has been increasing since the 1940s [1]. In Europe, climate change could favor the emergence of vector-borne emerging diseases by expanding the geographical distribution of vectors further north [2, 3] while international trade increases the chance of introducing vectors and pathogens. In animals, the emergence in northern Europe of the Bluetongue virus serotype 8 (BTV-8) in 2006 and of the Schmallenberg virus in 2011 illustrate this increasing risk in cattle, associated with the introduction of known pathogens in free areas or with the emergence of an unknown pathogen. Precise description of the disease effects and of their frequencies in exposed populations is needed to quantify diseases consequences and to refine surveillance. In the case of BTV8, clinical signs [4–6] as well as increased mortality [7–9] were described. The impact of BTV-8 on milk production [10, 11] and reproductive performance were quantified. BTV-8 was associated with both a decrease in fertility [12, 13] and an increased risk of abortion [14]. This impact on reproduction was due to the infection in pregnant cows (with a tropism of the virus in the genital tract) possibly followed by the infection of the fetus.

A recent study evaluated whether reproduction data could be used to build indicators suitable for syndromic surveillance [15]. This study showed an increased frequency of early calving (after a pregnancy length still within a normal range) in cows located in BTV infected areas, concomitant with the BTV notifications. Overall, an indicator based on the frequency of early calving was able to detect BTV outbreaks at an early stage. Given the short time interval between the notification of the first case and the occurrence of a detectable effect at a regional level, the delay between infection and the occurrence of an early calving should be short. Furthermore, because infections during early stages of gestation induced the most severe malformations [16], an event such as early calving in the normal range of gestation would be more likely associated with an exposure at late stages of gestation. Consequently, it is hypothesized that a viral infection at a late stage of pregnancy could trigger calving. No study has investigated the effect of BTV-8 infection for cows in late gestation on the frequency of early calving.

The surveillance system implemented in France was able to show the geographical progression of the BTV-8 epizootic but did not allow a precise estimation of its extent in terms of prevalence of infection [17]. This cross-sectional serologic study conducted in 2007 showed a high under-reporting rate. Moreover, a decrease in fertility was found for cows in herds located in the 2007 outbreak area but not reported as cases [18]. This decreased fertility should be at least partly due to the undetected or unreported viral circulation.

The objectives of this study were (1) to quantify the possible increase in frequency of early calving after a normal length of gestation associated with exposure to BTV-8 in late gestation and (2) to determine whether this association could be found in populations exposed to BTV-8 but without reported clinical signs.

## Materials and methods

### General study design and available data

Increases in the frequency of early calving were quantified for cows in herds reported as cases, based on the detection of clinical signs, as well as for cows in herds located in the 2007 outbreak area in France but not reported as cases. These frequencies were compared to frequencies of early calving in cows in herds not exposed to the virus.

**Figure 1:**
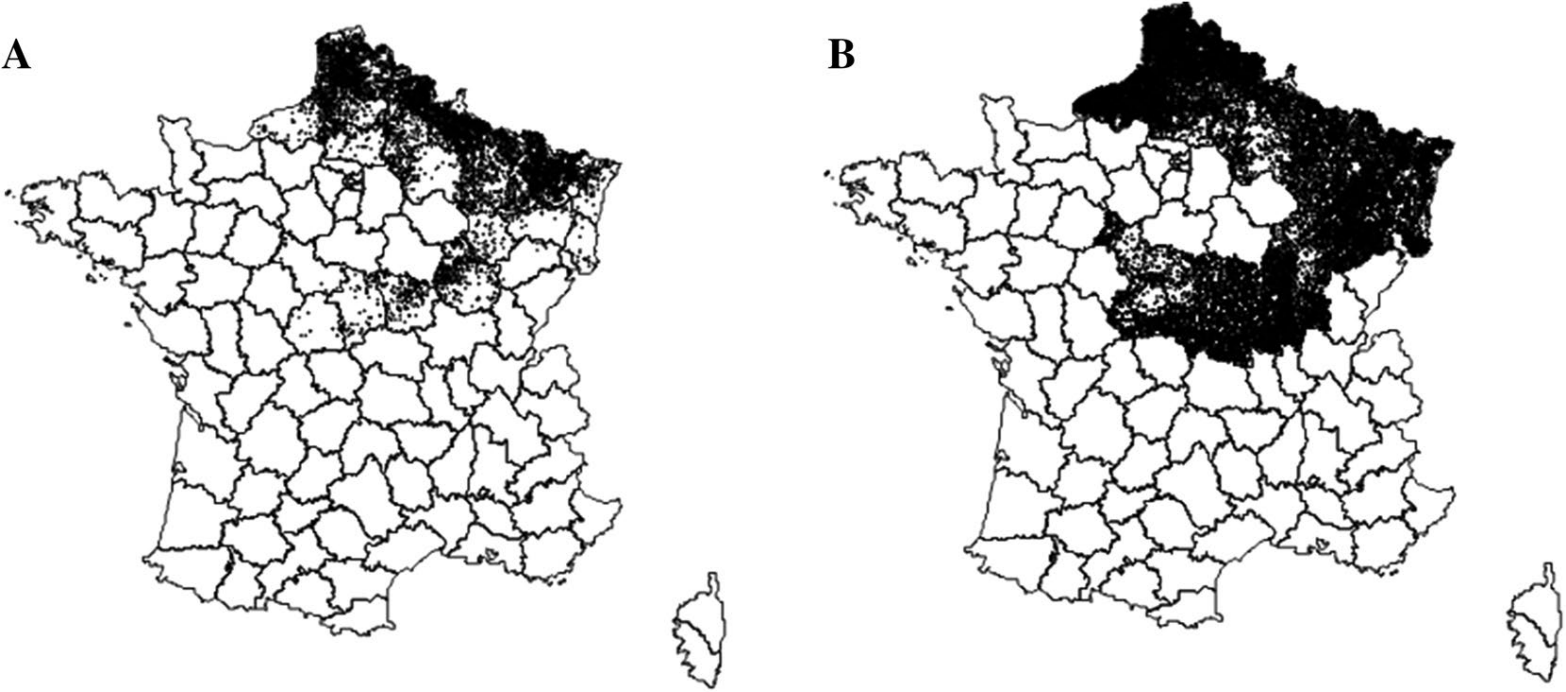
**Geographical location of cattle herds in the 23 departments located in the outbreak area.** **A** 8494 case herds reported after clinical signs of Bluetongue virus serotype 8 (BTV-8) in 2007, **B** 46 569 non-reported herds (e.g., herds with an interpolated date of BTV-8 exposure); 2007; France.

Information about notification of herds positive to BTV-8 during 2007 (from July to December) was obtained from the official veterinary surveillance system. Among herds reported during 2007, only herds with a confirmed detection reported after clinical suspicion were included. Information about BTV-8 exposure was available at the herd level only. Thus, all the cows of a herd were considered exposed if at least one animal with clinical signs had tested positive for BTV-8. The proportion of infected animals in reported herds was unknown. Herds selected for this study will be referred to as “case herds”. Case herds located in 23 departments (geographical and administrative unit) were selected (*n* = 8494), corresponding to 96% of all cattle herds reported with clinical signs during the 2007 epizootic (the non-selected case herds were located in departments with very low prevalence of BTV-positive herds). Cattle herds that were not reported during 2007 and located in these departments were considered as likely to have been exposed to BTV-8 (*n* = 46 569) (Figure 1). To see a description of the BTV notifications in France in 2007, see Durand et al. [17]. The geographical coordinates of herds were available at the municipality level.

The reproductive data were obtained from dairy herds where artificial insemination was used between October 2005 and September 2009. For each cow, obtained data were dates of AIs, date of culling (if it had occurred during the study period), calving date and data used to adjust for factors known to influence the length of gestation: cow and bull breeds and parity.

A single date of exposure to BTV-8 was estimated from recorded data for all the cows of a case herd. For herds located in the 2007 outbreak area but not reported as cases, a date of exposure was interpolated. This interpolation was based on the spatio-temporal dynamics of detection of confirmed case herds that reported clinical signs in 2007. Increase in early calving frequency was quantified in both case herds and herds located in the 2007 outbreak area but not reported as cases.

### Estimated dates of exposure for reported case herds

For each case herd, available data included the date at which clinical signs of disease were first suspected and the date at which disease was confirmed via diagnostic tests. Some of the cows were exposed earlier than the date of first suspicion since the BTV incubation period is between 2 and 18 days in cattle [19]. But not all the cows from a herd would be sick on the same day and clinical signs were considered to be associated with viremia in sick animals and could therefore serve as a source of virus for midges. Therefore, the estimated date of exposure for reported herds was defined as the recorded date of suspicion which corresponded to the first detection of clinical signs in the herd. The same date of exposure was assigned to all cows in a herd. For 6.1% of the case herds, the date of clinical suspicion was missing but the date of confirmation by a diagnostic test was known. In order to assign a date of suspicion, an imputation procedure based on the distribution of the time intervals between dates of suspicion and dates of confirmation was applied (values selected at random around a median interval of 4 days). Moreover, 181 case herds that had a non-plausible time interval between dates of suspicion and confirmation (interval >30 days or date of suspicion posterior to the date of confirmation) were excluded.

### Interpolation of dates of exposure for herds located in exposed areas but not reported as cases

**Figure 2:**
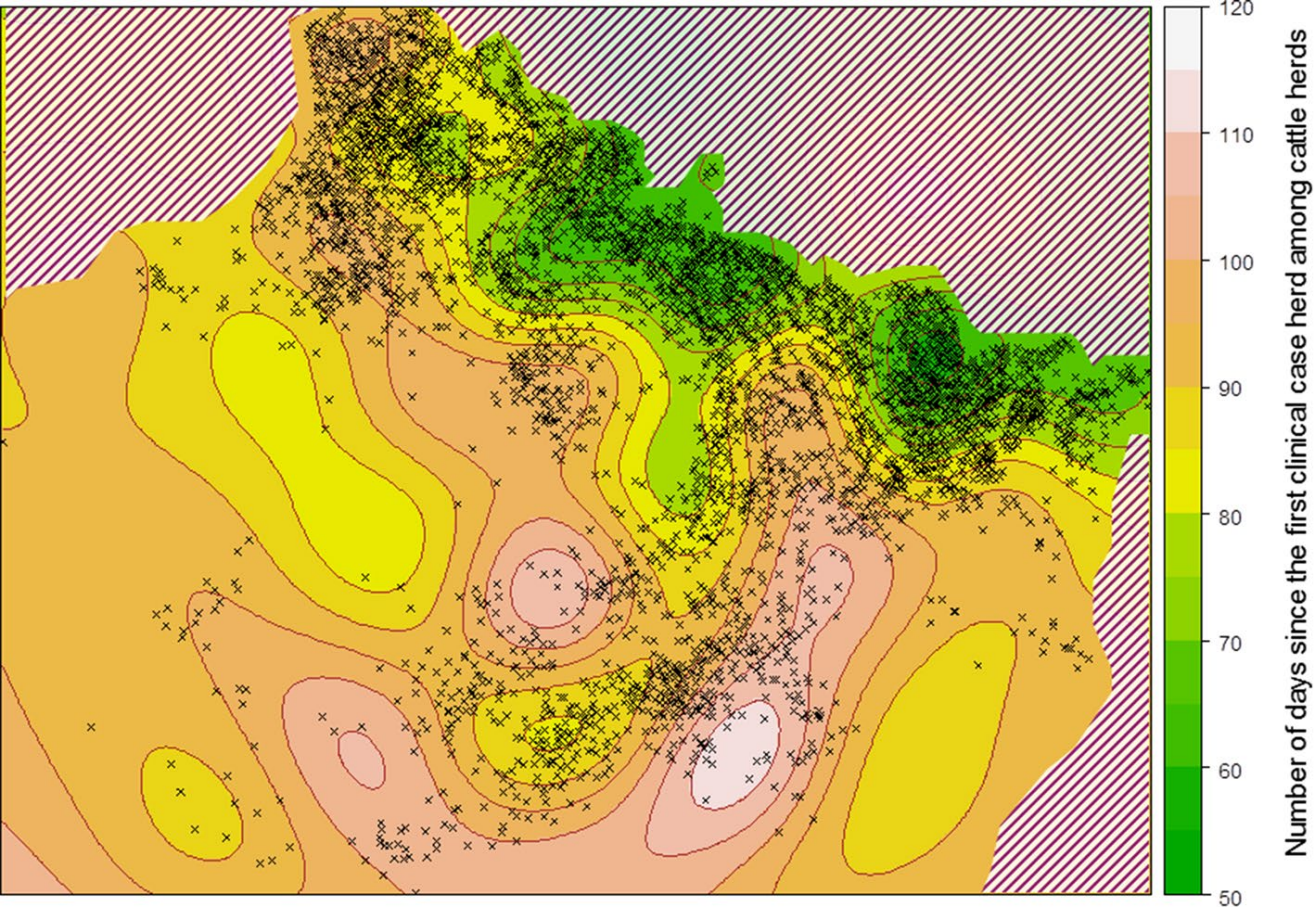
**Kriging map of the dates of exposure to Bluetongue virus serotype 8.** The dates were expressed as a number of days since the first clinical case herd during the 2007 epizootic in France (31^st^ July 2007), and location of reported case herds (black crosses). The hatched areas correspond to regions with no data (from [15]).

A date of exposure to BTV-8 was interpolated for each herd located in the 2007 outbreak area but not reported as case (Figure 2). Kriging, a geostatistical interpolation method, was used to estimate a date of exposure for these herds. Details on the method and on the steps followed can be found in Nusinovici et al. [18]. Dates were converted to numbers of days since the first case herd reported in 2007. Kriging uses a data sample (case herds) to predict values at unsampled locations (herd located in the 2007 outbreak area but not reported). All the cattle case herds (dairy and beef) were included as data samples because they could all have played a role in the virus spread and give a more precise picture of the epizootic wave diffusion than dairy herds alone. Kriging is based on assumptions regarding the form of the trend of the sample data, its variance and spatial correlation. The first step consisted in modelling the spatial correlation of the data. Two models were compared using a cross-validation process with observed data to determine each model’s goodness of fit and to compare their predictions. The final model was based on a Gaussian spatial component for filtering the random local component. Finally, to account for the non-stationarity of the BTV-8 spreading process, the gradient of the viral diffusion was also included in the model by the use of universal Kriging in place of ordinary Kriging.

### Selection of unexposed herds and cows

To test the robustness of results and to limit possible selection biases, two references unexposed populations were selected. The first reference population was composed of cows belonging to herds exposed in 2007 that had calved in 2005 and 2006 (before virus introduction in the area). The second was composed of cows belonging to herds situated in areas unexposed to BTV 1 and 8 in 2007, Britany and the Southwest of France (no other serotype was detected in continental France that year). These areas were chosen based on the geographical distribution of detected case herds in France in 2007 [17].

The month of calving is a known variation factor of gestation length [14, 20]. Cows in unexposed herds were therefore selected according to their calving date so that the cows in exposed (reported or not) and unexposed populations calved during the same period of the year.

### Definition of early calving and data selection

The last AI before calving was considered to be the insemination that led to conception. The gestation length was calculated as the interval between that AI and the date of calving. Early calving was defined as calving occurring between expected percentile 1 (LB for lower bound) and percentile 25 (HB for higher bound) of the normal gestation length. Expected percentiles were calculated with the Gaussian distributions of normal gestation lengths estimated using a sample of gestations between 260 and 320 days over a period without any major epidemics (from 2003 to 2005) [15]. Breed and parity influence the length of pregnancies. Therefore, LB and HB were calculated for each breed and each parity group (Additional file 1). Gestation lengths shorter than P1 (corresponding to 4.3% of the total number of gestations) were excluded because BTV-8 increases the risk of abortion which can be evidenced by very short gestation lengths (e.g., between 175 and 270 days [14]).

Data from the most common breeds in France, i.e., Holstein, Montbéliarde and Normande cows, were selected, representing 94.5% of the dataset. Cows inseminated with the semen of Holstein, Montbéliarde and Normande bulls were selected assuming that the gestation length could differ according to the bull’s breed (due to an effect of the calf weight). Cows with extreme or aberrant reproduction data were excluded: calving-first AI interval <35 or >180 days (corresponding to 1.8% of the total number of cows), assumed conceiving AI-calving interval >320 days (corresponding to 3.8% of the total number of cows). Herds under special management were also excluded (corresponding to 5.9% of the total number of herds): special demographic structure (% of primiparous cows within herds <10 or >75%), late breeding (calving-AI1 interval >120 days >75%) and herds likely to have used a bull (%AI2/AI1 < 10%). After these selections, populations of case herds, herds located in outbreak areas but not reported as cases and the two reference populations were composed of 2616, 4008, 11 038 (unexposed herds in 2007) and 8015 (herds in 2005/2006 located in the 2007 outbreak area) herds respectively.

### Selection and classification of cows according to the date of exposure within gestation

**Figure 3:**
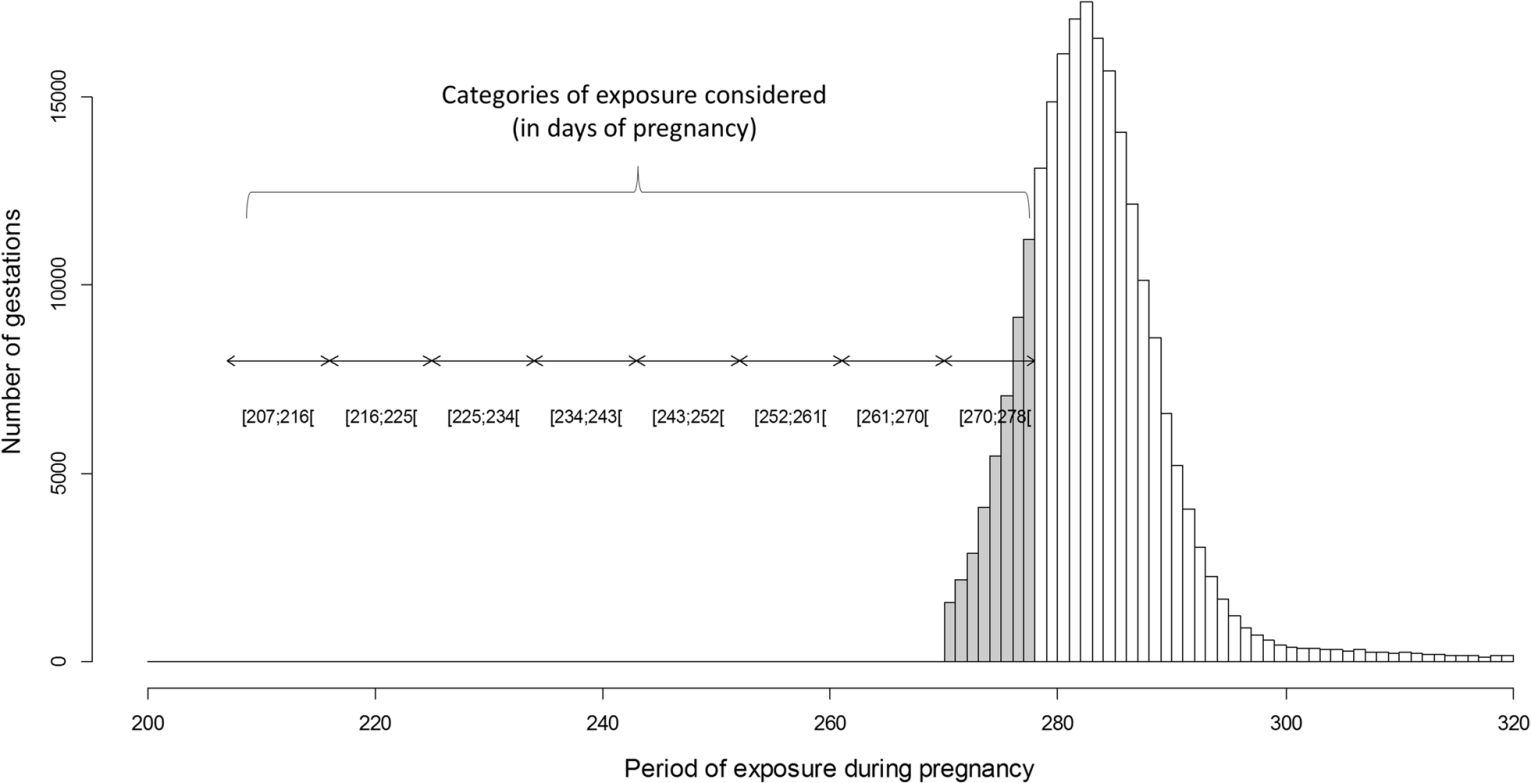
**Categories of exposure during gestation depending on the stage of pregnancy at the date of exposure (observed or interpolated).** The values considered corresponded to multiparous Holstein cows (time intervals for other breeds and parity in Additional file 1). The histogram represents the distribution of gestation lengths for multiparous Holstein cows, with the gray area corresponding to early calving (defined as the interval between the expected percentile 1 and 25 of the Gaussian distributions of normal gestation lengths).

The quantification of the BTV-8 exposure effect on the frequency of early calving took into account the date of exposure (observed or interpolated) within gestation. Only pregnancies exposed at the end of gestation were selected, from 63 days prior to LB to HB (exposures at HB were not included). Within this time interval, 8 categories of exposure were considered, corresponding to 9-day time intervals (except the last category LB-HB of 8-day length). The corresponding intervals in days of pregnancies for Holstein’s multiparous cows are detailed in the Figure 3 (for all intervals, see Additional file 1).

### Statistical models

The relationship between exposure to BTV-8 and occurrence of early calving was assessed using multivariable statistical models. To assess the frequency of early calving, a mixed-logistic regression model was used. To account for factors likely to influence the frequency of early calving, the association was adjusted for parity and month of calving as described by the following equation:

$$\begin{aligned} Y_{ij} \sim Bernouilli\,(p_{ij} ) \hfill \\ \log \left( {\frac{{p_{ij} }}{{1 - p_{ij} }}} \right) = \alpha + \beta_{1} EXP_{ij} + \beta_{2} PAR_{ij} + \beta_{3} MTH_{ij} + RANEF_{j} \hfill \\ RANEF_{j} \sim Normal\,\left( {0,\,\sigma^{2}_{j} } \right) \hfill \\ \end{aligned}$$

where the outcome Yij was a binary variable denoting the occurrence of an early calving in cow i from herd j, with a probability of occurrence pij; α was the intercept; EXPij was the exposure category. The exposure variable was categorized in 17 classes, depending on the time of exposure during pregnancy: eight classes corresponded to time intervals considered for cows with an observed date of exposure, eight classes corresponded to cows with an interpolated date of exposure and one class corresponded to the unexposed population (i.e., the reference population); PARj was the parity (4 classes: 1, 2, 3 and ≥4); MTHij was the month of calving (8 classes from August to March) and RANEFj was a random variable corresponding to the herd number. The random variable allowed adjusting for herd clustering. As odds-ratio (OR) overestimate the true relative risk (RR) when the incidence of the study event is high (frequency of early calving), OR were converted into RR using Beaudeau and Fourichon’s method [21]. The effect in percentage points of early calving frequency were calculated from RR estimates. Statistical analyses were performed using R software [22].

## Results

### Unadjusted frequencies of early calving

Raw frequencies of early calving in the two reference populations were comparable (Table 1). Without adjustment for parity and month of calving, frequencies were highest both for cows in case herds and cows in herds located in the outbreak area but not reported as cases.

**Table 1:** Number of herds, number of gestations and raw frequency of early calvings according to exposure statuses of the herd to Bluetongue virus serotype 8 (BTV-8)

	Number of herds	Number of gestations	Frequency of early calving (%)
Reported case herds with clinical signs in 2007	2616	17 139^a^	24.4
Non-reported herds located in the 2007 outbreak area	4008	20 139^a^	21.8
Unexposed herds in 2007	11 038	148 857^b^	19.5
Herds in 2005/2006 that were located in the 2007 outbreak area	8015	189 359	19.4

Early calving was defined as a calving after a pregnancy length between expected percentiles 1 and 25 of the Gaussian distribution of normal gestation length. 2005–2006: unexposed population, 2007: exposed population, France

^a^Gestations for which the date of herd exposure (observed or interpolated) occurred during the last 10 weeks of pregnancy (see “Materials and methods” section for more details).

^b^Gestations with calving occurring during the same period in the year than herds located in the outbreak area.

**Table 2:** Increase in frequencies of early calving, expressed as odds-ratios (OR) for cows in herds reported as cases of Bluetongue virus serotype 8 during the 2007 outbreak and cows in herds located in the 2007 outbreak area but not reported as cases

Type of herd	Category of exposure^a^	Reference population					
		Cows that calved in 2005 or 2006 belonging to herds located in the 2007 outbreak area			Cows in unexposed herds in 2007		
		Odds ratio	95% CI	*p* value	Odds ratio	95% CI	*p* value
Cows in case herds reported in 2007	(207–216)	1.06	0.94–1.21	0.34	1.10	0.97–1.26	0.14
	(216–225)	1.18	1.05–1.33	0.01	1.20	1.06–1.35	0.004
	(225–234)	1.25	1.11–1.40	<0.001	1.25	1.11–1.40	<0.001
	(234–243)	1.37	1.23–1.52	<0.001	1.37	1.23–1.53	<0.001
	(243–252)	1.51	1.36–1.66	<0.001	1.51	1.36–1.67	<0.001
	(252–261)	1.73	1.57–1.90	<0.001	1.70	1.54–1.88	<0.001
	(261–270)	1.81	1.65–1.98	<0.001	1.74	1.58–1.92	<0.001
	(270–278)	2.09	1.91–2.29	<0.001	1.97	1.79–2.16	<0.001
Cows in herds located in the 2007 outbreak area but not reported as cases	(207–216)	1.18	1.05–1.33	<0.001	1.30	1.15–1.46	<0.001
	(216–225)	1.27	1.14–1.42	<0.001	1.45	1.30–1.62	<0.001
	(225–234)	1.25	1.12–1.38	<0.001	1.43	1.28–1.60	<0.001
	(234–243)	1.30	1.18–1.44	<0.001	1.49	1.35–1.66	<0.001
	(243–252)	1.25	1.14–1.38	<0.001	1.39	1.25–1.53	<0.001
	(252–261)	1.23	1.12–1.35	<0.001	1.31	1.19–1.45	<0.001
	(261–270)	1.42	1.30–1.56	<0.001	1.47	1.34–1.61	<0.001
	(270–278)	1.34	1.22–1.47	<0.001	1.40	1.27–1.54	<0.001

In both populations, cows with dates of exposure (reported or interpolated) within the last 10 weeks of gestation were selected. The different OR values correspond to each category of exposure, defined according to the dates of exposure during pregnancy. Two reference populations were considered: cows that calved in 2005 or 2006 belonging to herds located in the 2007 outbreak area, and cows in unexposed herds on 2007 (Brittany and Southwest of France)

^a^The values considered corresponded to multiparous Holstein cows (for time intervals for other breeds and parity, see Additional file 1).

**Figure 4:**
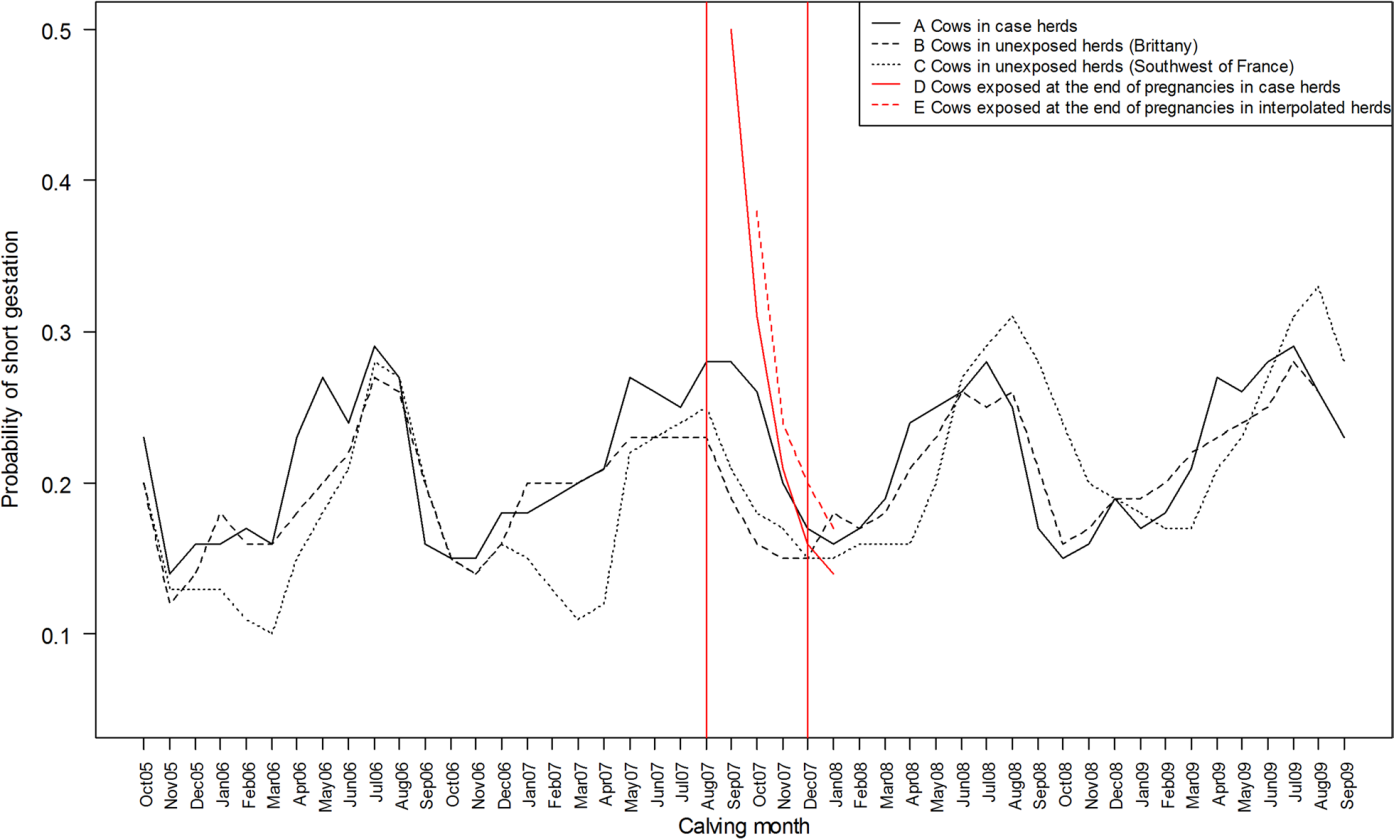
**Raw frequencies of early calving per calving month from October 2005 to September 2009.** (A) 34 248 cows in 2616 reported case herds with clinical signs in 2007; (B) 209 231 cows in 9540 unexposed herds located in Brittany; (C) 26 701 cows in 1498 unexposed herds located in the Southwest of France. B and C composed the unexposed population BTV-8 in 2007; (D) 17 139 cows in 2616 herds that reported clinical signs in 2007 exposed during the last 10 weeks of pregnancies. Population D is a subset of the population A. (E) 20 139 cows in 4008 herds for which a date of exposure was interpolated during the last 10 weeks of pregnancies. These herds were non-reported during the 2007 outbreak but located in the outbreak area. The two red vertical lines correspond to the 2007 outbreak period in France.

Except during the 2007 outbreak period, the frequency of early calving was quite stable over the years with a high seasonal effect (Figure 4). Frequencies of early calving of cows located in Brittany and Southwest of France showed similar patterns. When selecting only cows exposed between LB-63 days and HB, the increased frequency of early calving was much more pronounced (Figure 4, red curve). In the latter population, the highest increases were observed for calvings occurring in September and October 2007.

### Increased of early calving frequencies associated with BTV infection

**Figure 5:**
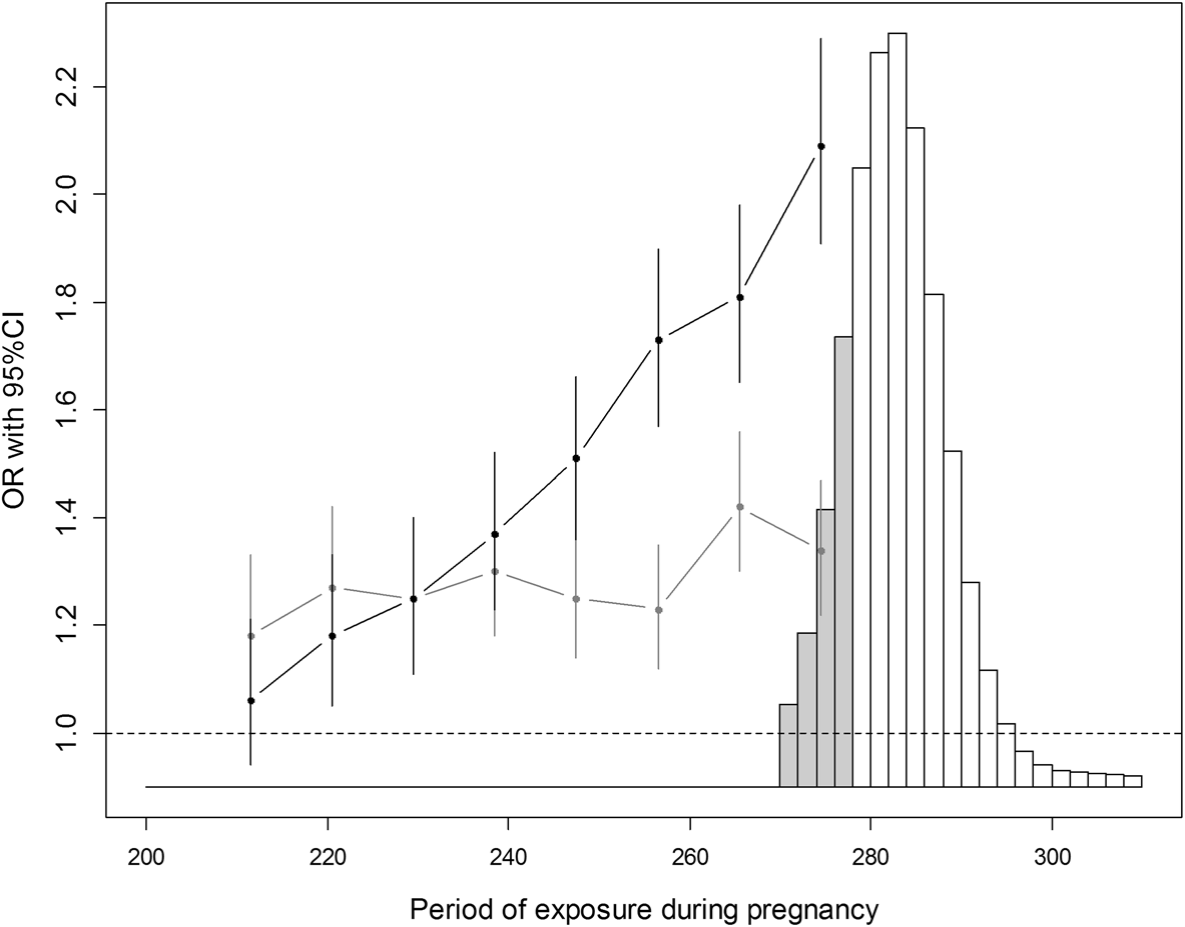
**Increase in frequencies of early calving.** The increases were expressed as odds-ratios (OR), for cows in herds reported as cases of Bluetongue virus serotype 8 during the 2007 outbreak (black curve) and cows in herds located in the 2007 outbreak area but not reported as cases (gray curve). In both populations, cows with dates of exposure (reported or interpolated) within the last 10 weeks of gestation were selected. The different OR values correspond to each category of exposure, defined according to the dates of exposure during pregnancy. The reference population considered was composed of cows that calved in 2005 or 2006 from herds located in the 2007 outbreak area. Vertical bars around ORs correspond to their 95% confidence intervals. The histogram represents the distribution of gestation lengths for multiparous Holstein cows, with the gray area corresponding to early calving (defined as the interval between the expected percentile 1 and 25 of the Gaussian distributions of normal gestation lengths).

Exposure to BTV-8 was associated with an increased frequency of early calving in both case herds and herds located in the outbreak area but not reported as cases (Figure 5). This increase was higher for exposures at the latest stage of pregnancy. The increase was higher for cows in case herds compared to cows in herds located in the outbreak area but not reported as cases. Results were similar whatever the reference population considered (Table 2). Exposure between day 270 and 278 yielded the highest OR (2.09 [1.91–2.29]), which corresponds to a RR of 1.73 [1.62–1.83]. Consequently, for 100 multiparous Holstein cows exposed between 270 and of 278 days of pregnancy, the frequency of early calving would be on average 33.6% [31.4–35.5], compared to 19.4% without exposure (corresponding to an average increase of 14.2%). The effects of parity and month of calving were significant (Table 3).

**Table 3:** Effect of adjustment variables on the frequency of early calving estimated with mixed logistic models

Variable and class	Number of pregnancies	Odds Ratio	95% CI	*p* value
Parity				
1	96 048	1	NA	
2	62 470	1.18	1.15–1.21	<0.001
3	37 036	1.11	1.08–1.15	<0.001
4 or more	37 293	1.10	1.07–1.14	<0.001
Month of calving				
August	17 525	1	NA	<0.001
September	24 525	0.59	0.57–0.62	<0.001
October	41 507	0.63	0.61–0.66	<0.001
November	52 305	0.49	0.47–0.51	<0.001
December	44 618	0.52	0.5–0.55	<0.001
January	26 490	0.51	0.48–0.53	<0.001
February	14 341	0.53	0.5–0.56	<0.001
March	11 536	0.57	0.54–0.61	<0.001

The unexposed population considered in this analysis corresponded to cows belonging to herds exposed in 2007 that calved in 2005 and 2006 (17 139 cows in 2616 case herds, 20 139 cows in 4008 herds located in the 2007 outbreak area but not reported and 189 359 cows in 8015 reference herds).

NA not applicable.

## Discussion

During the outbreak of 2007 in France, infection by BTV-8 in late gestation resulted in gestations that, although they were in the normal range of gestation lengths, were a few days shorter than expected. Increases in the frequency of early calvings were detected for both cows in herds reported as cases in 2007 and cows in herds located in the outbreak area but not reported as cases. For cows in case herds, increases in the frequency of early calving reached high values (OR > 2). To our knowledge, this is the first study quantifying the association of disease and triggering of an early calving. Such an effect would have been very difficult to detect with an experimental study for several reasons. Firstly, the event of interest lies within the normal/physiological range of variation. Indeed, the early calvings that were investigated in the present study would not qualify as abortions or any other pathological event to be recorded. Secondly, the time window between exposure to the virus and the observed increase in the frequency of early calvings was very narrow and the magnitude of the effect increased as the expected time to calving decreased. Thirdly, given the high influence of the season (in the same range of magnitude as the effect of exposure), an unbiased estimation of the effect would have required to stratify the observations by season, which would require a very large number of cows. The present study was carried out on a very large cattle population allowing a high statistical power. Moreover, the multivariable approach allowed controlling for known factors that influence gestation length. Finally, two reference populations were considered to limit possible bias because of variations in livestock management either between regions or over time. The results were similar regardless of the reference population. This robustness allows concluding that there was no major bias regarding variations in livestock management that would have not been accounted in the models.

The estimated magnitude of increased early calving frequency corresponds to mean effects in a herd under natural conditions of exposure in late gestation. In these herds, the proportion of infected cows was unknown. Therefore, these values likely underestimate the effect of infection for a single infected cow. BTV-8 is known to affect reproductive performances (fertility and abortion) due to infection in pregnant cows (with a tropism of the virus for the genital tract) possibly followed by the infection of the fetus. Interestingly, the effect of BTV-8 on early calving, for which no physiopathological mechanism is yet known, is higher in terms of magnitude of effect at a population level. However, lengths of pregnancies were comprised in a normal range and were expected to result in the birth of live calves. It can therefore be expected that the economic impact at the herd level may be low.

The highest effect of BTV-8 infection was found for exposures occurring during the latest stage of pregnancy. The impact of exposure to BTV on the mechanism triggering calving could therefore be higher in the very last stage of gestation compared to earlier exposures. This finding is in accordance with Marceau et al. [15] that indicated that delay between infection and the occurrence of an early calving should be short. Consequently, cows infected at the latest stage of pregnancy calve a few days earlier than expected i.e., that the infection by BTV-8 triggers calving. Increases in early calving were also observed for exposures from 200 days of gestation. Because the dates of infections of cows within these herds are unknown, it is difficult to conclude about effects associated with these early exposures. On the one hand, this could be related to the time needed for the virus to spread within herds: due to this infection dynamic, some cows within infected herds could have been infected weeks after the date of first infection. On the other hand, this could be due a delayed effect of infection, but no results are available to support or infirm this hypothesis.

An increase in the frequency of early calving was quantified in herds located in the outbreak area but not reported as cases. This effect corresponded to approximately half of the increase quantified in herds reported with clinical signs. A similar proportion was found for the quantification of fertility decrease in herds located in the outbreak area but not reported compared to the effect of BTV-8 in case herds [18]. Because of the high under-reporting rate, some of the herds located in the outbreak area but not reported have probably been infected during the outbreak. Infected herds could have not been reported as cases either because no animal showed any clinical signs (asymptomatic BTV-8 infections in cattle were quite frequent [4, 23]) or because the clinical signs were not specific and thus not attributed by farmers to BTV-8 infection.

It has been demonstrated before that an indicator based on short gestation was able to detect the 2007 BTV outbreak at an early stage with a large number of abnormal elevations in infected areas [15]. The present study strengthens the interest of considering such an indicator for the following reasons. Firstly, the magnitude of the increased early calving frequency associated with BTV-8 infection was high. It is known that the ability to detect a disease using syndromic surveillance is highly related to the magnitude of its effect on the indicator used [24]. The related biological mechanism behind early calving is unknown. This event is different from an abortion because it occurs within the normal range of gestation lengths and is expected to result in the birth of live calves. It would have been interesting to investigate whether other diseases are associated with such increases frequency of early calving. If so, that would confirm the interest of considering early calving as an indicator for syndromic surveillance. Secondly, increased frequency of early calvings was detected in areas with infected herds that were not reported through a clinical surveillance system. Infections with very moderate clinical signs or clinical signs not specific to BTV-8 were frequent in cattle during the epizootic in North–west Europe [4, 6]. That suggests that early calving could be associated with non-specific biological phenomena such as fever in infected cows, as suggested by [15]. An indicator associated with non-specific symptoms would allow detecting diseases that are particularly difficult to detect through clinical surveillance. Moreover, such symptoms might be associated with a wide range of infectious diseases. Finally, on the contrary to other effects of BTV-8 on reproductive performance (increase embryonic mortality and abortion), the increase in early calving should occur quickly after infection. Using this indicator for syndromic surveillance could therefore contribute to the early detection of pathogens with such an effect on reproductive performance.

## Additional file

10.1186/s13567-015-0296-7 **Time intervals considered for each category of exposure depending on breed and parity.** This additional file contained the time intervals considered for each category of exposure depending on breed and parity. These time intervals varied depending on breed and parity because these factors influence the length of pregnancies.
