# Supplementary material for: Quantification of the increase in the frequency of early calving associated with late exposure to bluetongue virus serotype 8 in dairy cows: implications for syndromic surveillance
Source: Vet Res. 2016 Jan 13;47:18. doi: 10.1186/s13567-015-0296-7 (PMC4711031; doi:10.1186/s13567-015-0296-7)
Supplement: Supplementary file 1 — 10.1186/s13567-015-0296-7 Time intervals considered for each category of exposure depending on breed and parity. This additional file contained the time intervals considered for each category of exposure depending on breed and parity. These time intervals varied depending on breed and parity because these factors influence the length of pregnancies. [file 13567_2015_296_MOESM1_ESM.docx]

**Additional file 1 Time intervals considered for each category of exposure depending on breed and parity.**

|  | Exposure category 1 | Exposure category 2 | Exposure category 3 | Exposure category 4 | Exposure category 5 | Exposure category 6 | Exposure category 7 | Exposure category 8 |
| --- | --- | --- | --- | --- | --- | --- | --- | --- |
| Time interval | [P1-63; P1-54[ | [P1-54; P1-45[ | [P1-45; P1-36[ | [P1-36; P1-27[ | [P1-27; P1-18[ | [P1-18; P1-9[ | [P1-9; P1[ | [P1; P25[ |
| Holstein - primiparous | [205; 214[ | [214; 223[ | [223; 232[ | [232; 241[ | [241; 250[ | [250; 259[ | [259; 268[ | [268; 277[ |
| Montbéliarde - primiparous | [211; 220[ | [220; 229[ | [229; 238[ | [238; 247[ | [247; 256[ | [256; 265[ | [265; 274[ | [274; 283[ |
| Normande - primiparous | [210; 219[ | [219; 228[ | [228; 237[ | [237; 246[ | [246; 255[ | [255; 264[ | [264; 273[ | [273; 282[ |
| Holstein - mutiparous | [207; 216[ | [216; 225[ | [225; 234[ | [234; 243[ | [243; 252[ | [252; 261[ | [261; 270[ | [270; 278[ |
| Montbéliarde - mutiparous | [211; 220[ | [220; 229[ | [229; 238[ | [238; 247[ | [247; 256[ | [256; 265[ | [265; 274[ | [274; 283[ |
| Normande - mutiparous | [211; 220[ | [220; 229[ | [229; 238[ | [238; 247[ | [247; 256[ | [256; 265[ | [265; 274[ | [274; 282[ |
| Number of pregnancies in case herds with clinical signs | 1956 | 2207 | 2358 | 2500 | 2621 | 2730 | 2734 | 2465 |
| Number of pregnancies in herds not reported located in the 2007 outbreak area | 3296 | 3747 | 3941 | 4441 | 4849 | 5191 | 5372 | 4876 |

P1 and P25: percentile 1 and percentiles 25 of the Gaussian distributions of normal gestation lengths. These percentiles were estimated for each breed and parity group using a sample of gestations between 260 and 320 days over a period without any major epidemics (from 2003 to 2005).
